# Critical closing pressure of the pharyngeal airway during routine drug-induced sleep endoscopy: feasibility and protocol

**DOI:** 10.1152/japplphysiol.00624.2021

**Published:** 2022-02-03

**Authors:** Elahe Kazemeini, Eli Van de Perck, Marijke Dieltjens, Marc Willemen, Johan Verbraecken, Scott A. Sands, Olivier M. Vanderveken, Sara Op de Beeck

**Affiliations:** ^1^Translational Neurosciences, Faculty of Medicine and Health Sciences, University of Antwerp, Wilrijk, Belgium; ^2^ENT, Head and Neck Surgery, Antwerp University Hospital, Edegem, Belgium; ^3^Multidisciplinary Sleep Disorders Centre, Antwerp University Hospital, Edegem, Belgium; ^4^Laboratory of Experimental Medicine and Pediatrics, Faculty of Medicine and Health Sciences, University of Antwerp, Wilrijk, Belgium; ^5^Department of Pulmonology, Antwerp University Hospital, Edegem, Belgium; ^6^Division of Sleep and Circadian Disorders, Brigham and Women's Hospital and Harvard Medical School, Boston, Massachusetts

**Keywords:** collapsibility, endotyping, DISE, obstructive sleep apnea, upper airway

## Abstract

In obstructive sleep apnea (OSA), there are various pathophysiological factors affecting the upper airway during sleep. Two prominent factors contributing to OSA are site and pattern of upper airway collapse and degree of pharyngeal collapsibility. In a clinical setting, drug-induced sleep endoscopy (DISE) is used to visualize the structures of the upper airway. Critical closing pressure (Pcrit) is the gold standard measure of pharyngeal collapsibility. This prospective clinical study aimed to investigate the feasibility and protocol of Pcrit measurements during DISE. Thirteen patients with OSA were included. Pcrit was calculated using peak inspiratory airflow and inspiratory ventilation. The proposed protocol was successful in Pcrit measurement during DISE in all subjects [median[Q1;Q3] Pcrit for “peak inspiratory method” (*n* = 12): −0.84[−2.07;0.69] cmH_2_O, “ventilation method” (*n* = 13): −1.32[2.32;0.47] cmH_2_O], highlighting the feasibility of the approach. There was no significant difference (*P* = 0.67) between calculated Pcrit with either of the calculation methods, indicating high reliability. Correlation analysis showed Pcrit as an independent parameter of any of the anthropometric or polysomnographic parameters. The ventilation method proved to be more successful in assessment of Pcrit in subjects with epiglottic collapse (e.g., with high negative effort dependence). Subjects with palatal complete concentric collapse during DISE had a wide Pcrit range ([−2.86;2.51]cmH_2_O), suggesting no close correlation between Pcrit and this DISE pattern (*P* = 0.38). Incorporation of Pcrit measurements into DISE assessments is feasible and may yield valuable additional information for OSA management. Combining Pcrit and DISE provides information on both the site and degree of upper airway collapse and the degree of pharyngeal collapsibility.

**NEW & NOTEWORTHY** The protocol of this study was successful in concomitant measurement of Pcrit during routine clinical endoscopy. Comparison of two calculation methods for Pcrit showed that the inspiratory ventilation method was more successful in assessment of Pcrit in subjects with epiglottic collapse who have high negative effort dependence. Subjects with palatal complete concentric collapse during DISE had a wide Pcrit range and did not have a greater Pcrit than patients in other site of collapse categories.

## Graphical abstract

**Figure:**
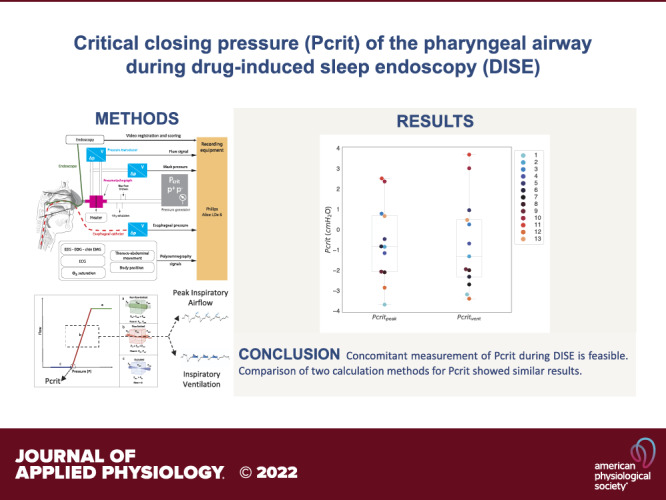

## INTRODUCTION

Within the spectrum of sleep-disordered breathing, obstructive sleep apnea (OSA) is highly prevalent (1). OSA is caused by recurring collapse of the upper airway during sleep, resulting in complete (apnea) or partial (hypopnea) cessation of airflow (2). OSA severity is expressed by the apnea-hypopnea index (AHI), defined as the number of apneas and hypopneas per hour of sleep (3).

The standard noninvasive treatment of OSA is continuous positive airway pressure (CPAP), which creates a pneumatic splint in the pharyngeal airway (4). When CPAP is used at the optimal pressure, it has high efficacy in eliminating OSA and improving OSA-related health outcomes in nearly everyone regardless of the underlying pathophysiology (5). However, the effectiveness of CPAP might be hampered by a rather limited patient adherence and tolerability (6). This raises the need for other non-CPAP therapies. There are various non-CPAP options available that affect the anatomical structure of the upper airway such as mandibular advancement device (MAD) or different surgical modalities, yet efficacy of these treatments is patient-dependent. Therefore, understanding the underlying pathogenesis of OSA is important in upfront patient selection for these non-CPAP therapies (7–9).

In patients with OSA, various pathophysiological factors affect the patency of the upper airway and the amount of ventilation during sleep. Two prominent factors that contribute to OSA are the site and pattern of collapse and the collapsibility of the upper airway (10, 11).

Sleep endoscopy is used to visualize the structures of the upper airway. It is a crucial diagnostic tool for OSA that provides information about the level, degree, and configuration (pattern/direction) of upper airway collapse (12, 13). It can either be performed during natural sleep or during drug-induced sleep (13, 14). Due to the time-consuming and labor-intensive nature of natural sleep endoscopy, drug-induced sleep endoscopy (DISE) is preferred in clinical practice, as it can be performed during a short period of time and within normal daytime working hours (12, 13). The DISE site, pattern, and degree of upper airway collapse were shown to be associated with the therapeutic outcome of several non-CPAP treatment options (8, 15–17). Especially complete concentric collapse at the level of the palate (CCCp) is an important patient selection criterion. It is a formal exclusion parameter for hypoglossal nerve stimulation and is associated with adverse MAD treatment outcomes (8, 16).

Critical closing pressure (Pcrit) is the gold standard measure of collapsibility of the pharyngeal airway (18). Pcrit is an essential part of categorizing subjects with OSA into various endotypic groups, which subsequently improves treatment and response prediction (19).

**Figure 1.**
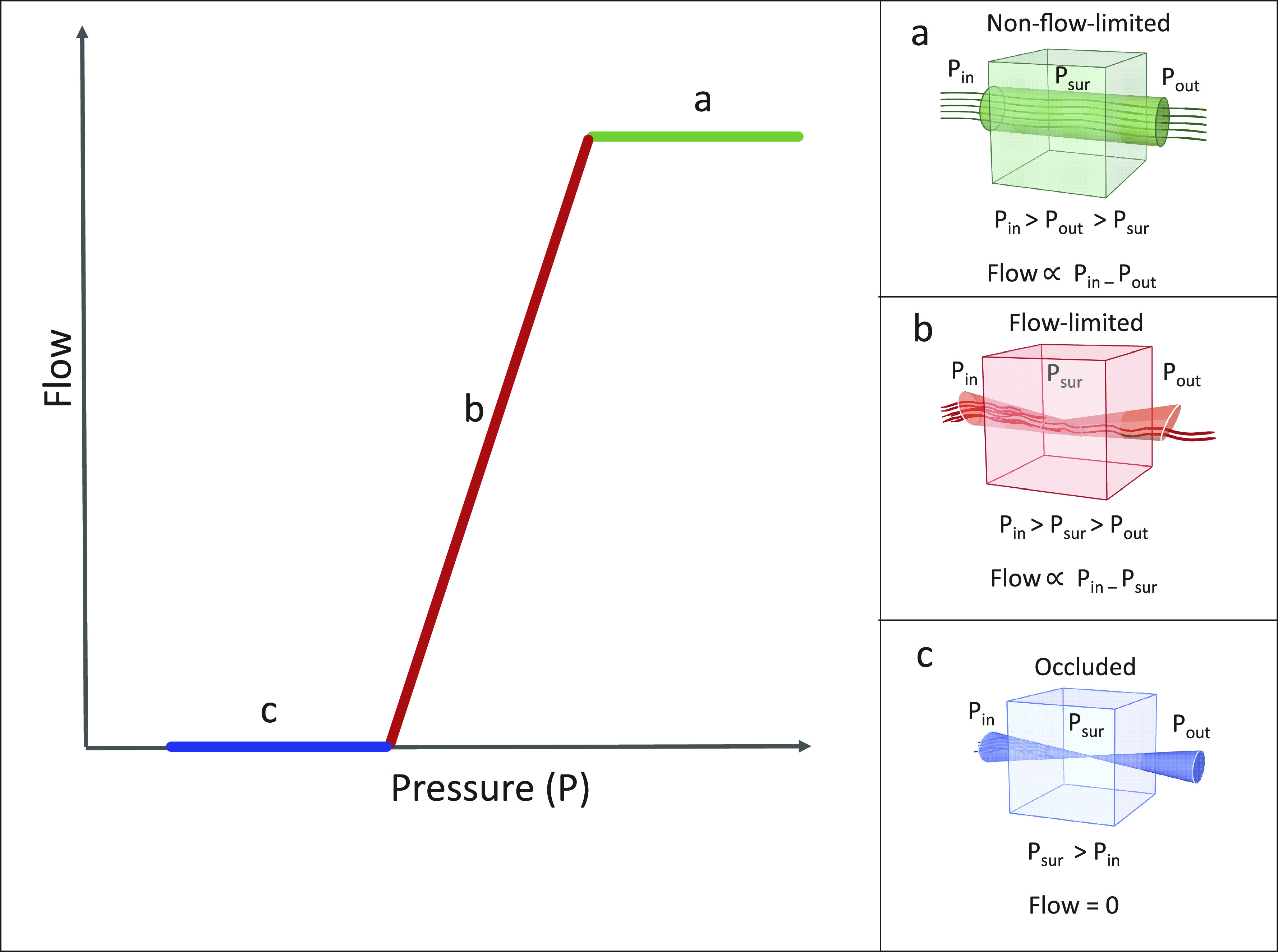
Schematic model of the pressure-flow relationship. Pin, inflow pressure; Pout, Outflow pressure; Psur, surrounding pressure.

Various methods for Pcrit measurement during sleep have been introduced. The core of most of these methods is to increase the pressure to a level where the pharyngeal airway is open and airflow is not limited (holding pressure, Fig 1*A*). Then, multiple series of pressure drop (Fig 1*B*) are performed to reach a pressure at which the airflow is zero (Fig 1*C*). Thereafter, the pressure-flow relationship is calculated using linear regression to determine Pcrit (Fig. 1) (20–22). Two analysis methods for Pcrit calculations after data acquisition are used *1*) using peak inspiratory flow and *2*) using inspiratory ventilation. Using these techniques of pressure drop, both the “active Pcrit” and the “passive Pcrit” can be determined. If the pressure is lowered slowly, allowing for dilator muscles of the upper airway to activate, the measured critical closing pressure, is called “active Pcrit” (23). To measure “passive Pcrit,” the pressure is dropped abruptly to record flow before the dynamic muscle activation (23).

The gold standard method of measuring Pcrit is usually applied during natural sleep, making it cumbersome and, for similar reasons as with natural sleep endoscopy, not feasible in routine clinical practice. Therefore, several studies have sought surrogate parameters for this pathophysiological trait (7, 24–27). The upper airway collapsibility index, as measured by application of negative pressure pulses in brief periods during wakefulness, showed to have a strong association with Pcrit and might be a viable marker of the pharyngeal collapsibility level (25). Furthermore, according to Azarbarzin et al. (27), peak inspiratory flow at atmospheric pressure might reflect Pcrit, offering the possibility to estimate the level of collapsibility directly from baseline diagnostic airflow signals.

The main challenges of the gold standard method of Pcrit measurement during natural sleep are multiple arousals, its time-consuming nature and the need for long overnight studies. Measuring Pcrit during drug-induced sleep overcomes the problem of recurrent arousals and reduces time requirements. Studies have shown that collapsibility levels during midazolam sedation are similar to that of natural sleep (28). Having data on the level of collapsibility and the structural pattern of collapse at the same time can be a step toward an advanced personalized OSA management (7, 29, 30). Pcrit under direct visualization has been previously measured in a research setting (30). However, no previous study has investigated the feasibility and protocol of Pcrit measurements during routine clinical DISE allowing concomitant Pcrit measurement and endoscopy. As a primary outcome, the current study aimed to assess the feasibility of Pcrit measurements during routine clinical DISE and to develop a feasible protocol. Two different methods of Pcrit calculation, one using peak inspiratory airflow (Pcrit_peak_) and the other using inspiratory ventilation (Pcrit_vent_), were compared. As a secondary outcome, association between the site of upper airway collapse and collapsibility level were researched. In addition, pressure-flow relationship outcomes including upstream resistance (Rus), median of the minimally effective CPAP level at which airflow limitation was first abolished and median level of the nasal pressure at which inspiratory airflow was first ceased as observed during the measurements (Pclose-observed) were also investigated.

## METHODS

This study was designed as a prospective feasibility clinical trial. Patients with an established OSA diagnosis (AHI ≥15/hour sleep) who were eligible for DISE as the next step in their clinical pathway, were eligible and recruited for this study. All patients provided written informed consent. The study was reviewed and approved by the local ethics committee at Antwerp University Hospital and the University of Antwerp and registered at clinicaltrials.gov (NCT04232410).

### Pcrit Set-up and Measurement

#### Set-up.

Patients underwent a standard clinical DISE with additional measurements. Polysomnography (PSG) signals including electroencephalography (EEG), chin electromyography (EMG), electrooculography (EOG), oximetry, and thoracoabdominal movements were recorded. All subjects were fitted with a modified nasal mask (ComfortGel Blue, Philips Respironics) connected to a heated pneumotachometer (Hans Rudolf, Kansas City, MO) and a differential pressure transducer for measurement of airflow and pressure of the mask (Pmask). Air leaks through the mouth were avoided by the use of surgical tape. Signals were recorded through a PSG system (Philips Alice LDx 6) via data acquisition software (Sleepware, Philips Respironics). Each mask had modified holes for endoscopy that were sealed with exercise putty (CanDo TheraPutty, Black X-firm) after insertion of the flexible fiberoptic nasopharyngoscope (Olympus END-GP, 3.7 mm diameter, Olympus Europe GmbH, Hamburg, Germany) to avoid air leak. A balloon esophageal catheter [esophageal balloon catheter 5 French (1.6 mm), Cooper surgical] was inserted through one of the nostrils into the esophagus and its signal was recorded via a separate differential pressure transducer.

**Figure 2.**
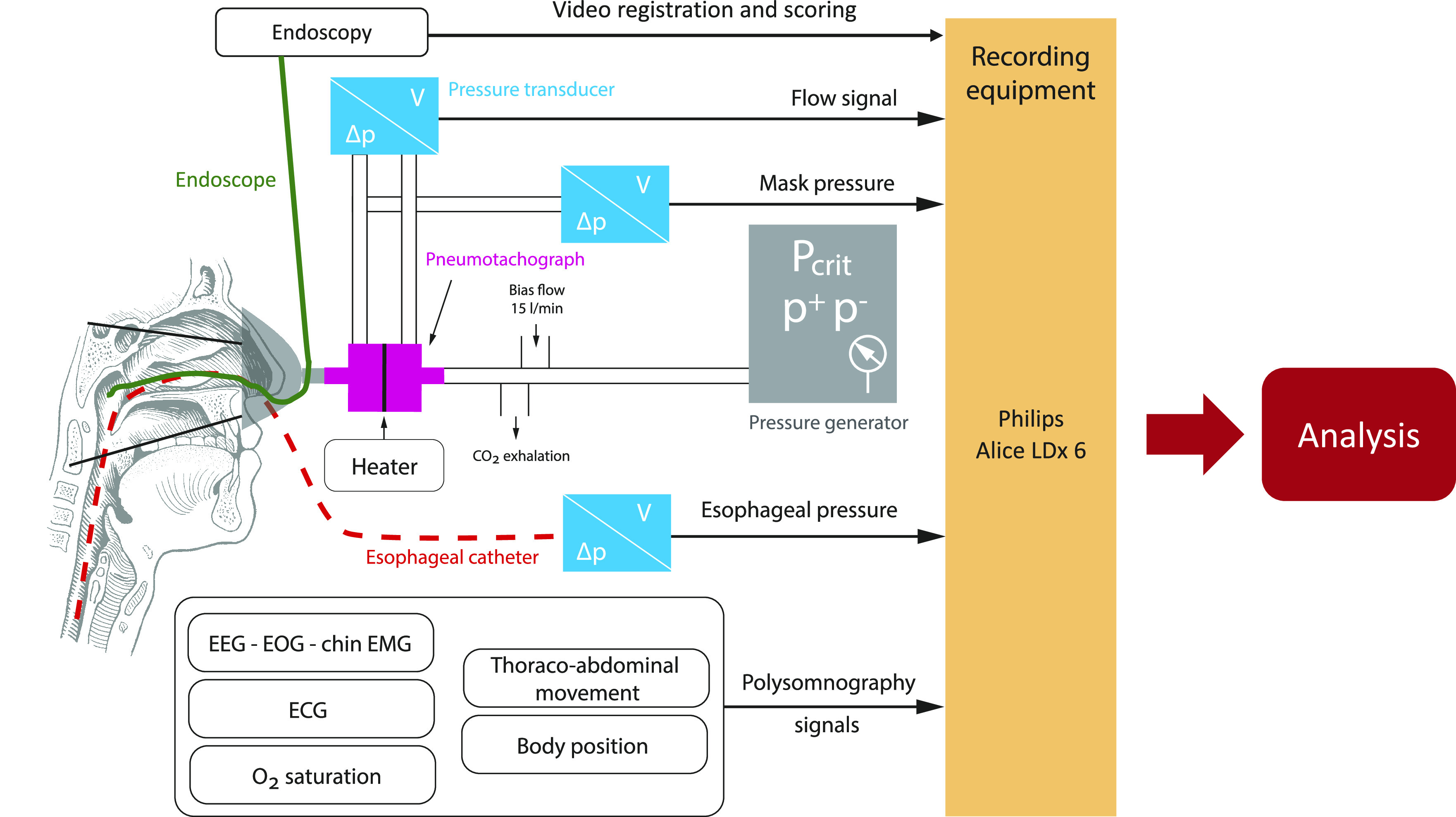
Schematic overview of set-up for measurement of critical closing pressure (Pcrit) of the pharyngeal airway during drug-induced sleep endoscopy (DISE).

A modified CPAP device (Pcrit3000, Philips Respironics), capable of producing both negative and positive pressures between the range of −20 to +20 cmH_2_O, was used. In addition, a few safety measures were implemented: to minimize cross infection, a bacterial filter was installed at the outlet of the CPAP device. To avoid CO_2_ rebreathing, a whisper swivel was added after the pneumotachometer and a bias flow of 12 L/min was administered through an O_2_ enrichment piece. This O_2_ enrichment piece was connected to the CPAP device via a CPAP tube (Fig. 2).

#### Pcrit measurement.

DISE was performed in a semidark, silent operating theater with the patient lying in supine position. For the induction of sleep, a single IV bolus of midazolam (1.5 mg) was administered. Subsequently, propofol (2.0–3.0 µg/mL) was administered via a target-controlled infusion (TCI) pump. After insertion of the endoscope and confirmation of sleep via EEG signals and absence of air leak check, the Pcrit measurement procedure was started. The first step was to determine a “holding pressure,” a pressure at which there is no airflow limitation. The CPAP pressure was gradually increased to eliminate all flow limitations. This level of pressure was used as the individual “holding pressure” for each subject. The holding pressure was primarily determined by flow shape while considering esophageal pressure swings.

**Figure 3.**
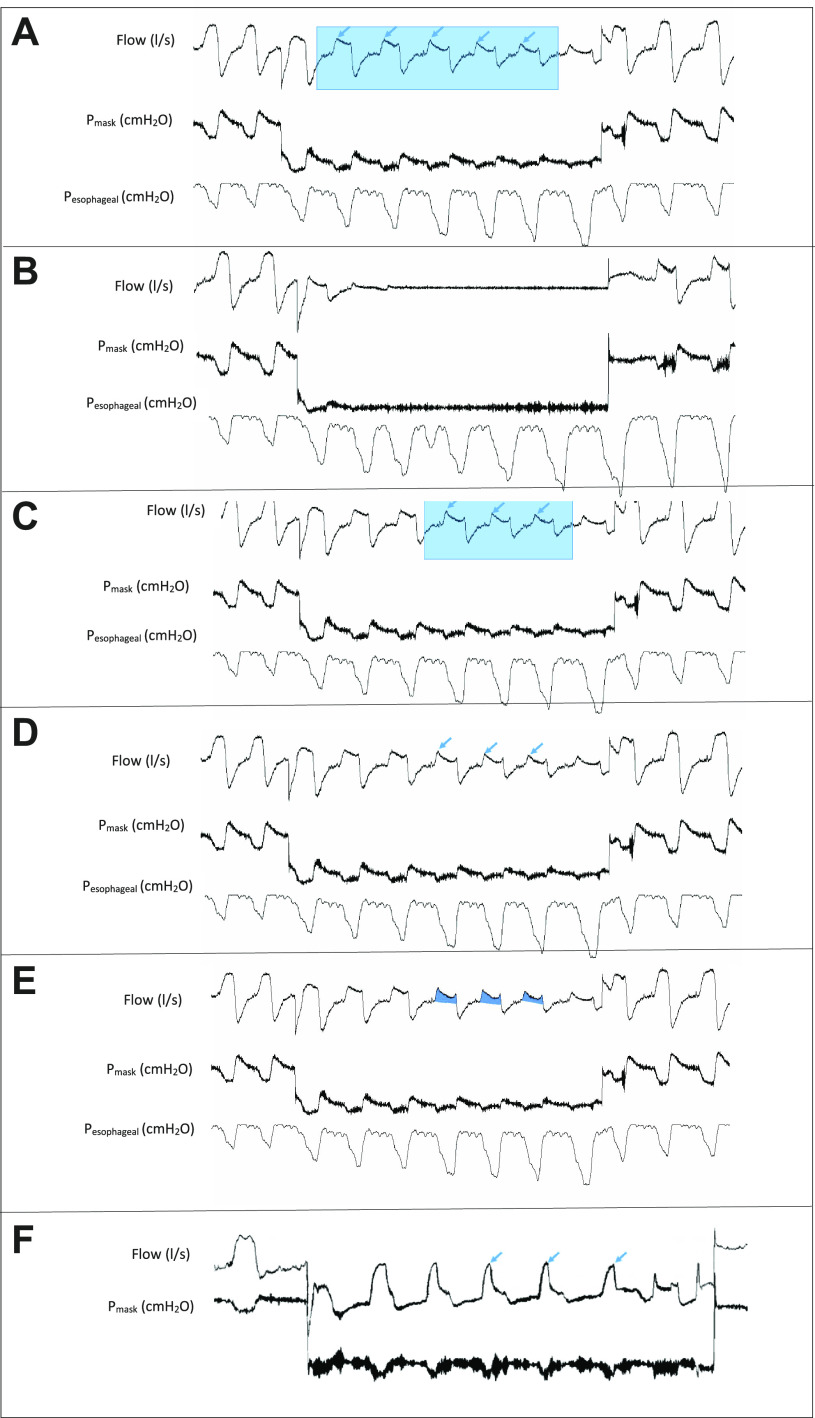
Sample representatives of flow with a pressure drop for five breaths. *A*: start and endpoints of each 5-breath pressure drop (run) were determined by expert visual analysis. *B*: sample of an apneic event as a result of a pressure drop. *C*: breaths 3 to 5 of the pressure drop were used in the analysis. *D*: maximum inspiratory airflow (arrows) was used in the calculations with peak inspiratory method. *E*: inspiratory ventilation for flow-limited breaths 3 to 5 was used in calculations with the ventilation method. *F*: sample breaths with airflow pattern pointing to the presence of high negative effort dependence (NED), reflected by the high peak at the start of inspiration (arrows) followed by a low plateau inspiration. Mask pressure (P_mask_), pressure recorded via balloon esophageal catheter (P_esophageal_).

Thereafter, the CPAP pressure was abruptly dropped from the holding pressure by 2 cmH_2_O for the duration of five breaths (one run). Subsequently, the pressure was set back to the holding pressure (Fig. 3*A*). This pressure drop procedure was repeated in increments of 2 cmH_2_O until an apnea was observed. Upon observation of apnea, the pressure was set to 1 cmH_2_O higher than the last used pressure to perform the final pressure drop. This pressure drop process was replicated for up to three series in each subject.

The scoring of sleep endoscopy was based on the level (palate, lateral pharyngeal walls, tongue, epiglottis, and hypopharynx), degree (complete, partial, and none), and configuration (anteroposterior, latero-lateral, and circular) of the upper airway collapse. The tip of the endoscope was placed at the base of the tongue and right before the epiglottis, during the “holding pressure” assessment. The first series of pressure drop was performed with the endoscope still in the same position. Afterward, for the second series of pressure drop, the endoscope was retracted to right after the palate to the level of the tonsils. During the third series of pressure drop, the tip of the endoscope was positioned right before the palate in the nasopharynx.

### Pcrit Calculation

Pcrit was calculated by two different methods as follows:

#### Peak inspiratory method (Pcrit_peak_).

The first analysis step was to extract the airflow from the recordings. Start- and endpoints of each 5-breath pressure drop (run) were determined by expert visual analysis of the flow signal (Fig. 3, *A* and *B*). Flows from breaths 3 to 5 of the pressure drop were used in the analysis (Fig. 3*C*). Peak inspiratory flow was defined as:

Peak inspiratory flow = maximum inspiratory airflow (Fig. 3*D*) − average airflow over the span of the 5-breath pressure drop (Fig. 3*A*)

**Figure 4.**
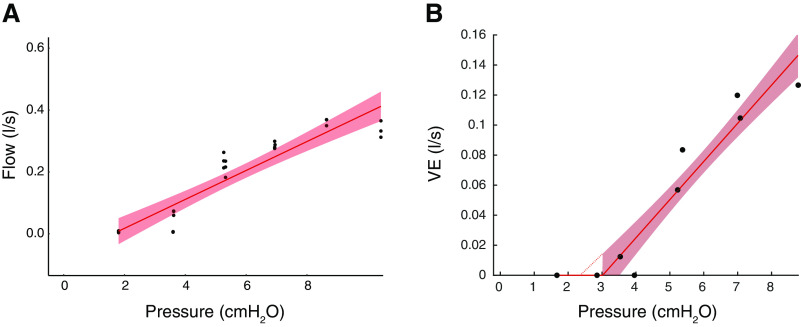
Sample representatives of pressure-flow linear regression of *subject 10* for both calculation methods. *A*: peak inspiratory method sample: Pcrit was determined as the zero-flow intercept from the linear regression of flow vs. pressure of the isolated segment between Peff and near Pcrit points (Pcrit = 2.36 cmH_2_O). *B*: V̇e, minute ventilation converted to l/s, ventilation method sample (Pcrit = 3.01 cmH_2_O). Pcrit, critical closing pressure; Peff, upper inflection point.

The peak inspiration was calculated separately for breaths 3 to 5. Subsequently, all breaths were plotted against their corresponding Pmask. Based on this plot and the method previously explained by Patil et al. (22), an upper inflection point (Peff), the theoretical ideal holding pressure, which is the lowest pressure above which airflow is no longer limited and below which airflow is limited, and a lower inflection point (near Pcrit) were identified (Fig. 4*A*). Hereafter, the range of pressure between these two inflection points, over which the flow varied markedly (Fig. 1*B*), was isolated. This isolated range was used to calculate Pcrit (22). Pcrit was determined as the zero-flow intercept from the linear regression of flow versus Pmask of this isolated segment (Fig. 4*A*).

#### Ventilation method (Pcrit_vent_).

In this method, the inspiratory ventilation for flow-limited breaths three to five of each pressure drop was manually extracted using a custom-designed software (MATLAB, The MathWorks, Natick, MA, 2019b) (Fig. 3*E*). Median ventilation of each flow-limited breath was calculated. Outlier data were manually removed based on expert visual analysis. Thereafter, Pcrit was determined as the zero-flow intercept from the linear regression of ventilation versus Pmask (Fig. 4*B*).

### Statistical Analysis

Statistical analysis was conducted using *R* software (R Foundation for Statistical Computing, Vienna, Austria, Version 1.4.1106), Python (Python software foundation, Centrum voor Wiskunde en Informatica, Amsterdam, Netherlands, version 3.9. 0), and MATLAB (MATLAB, The MathWorks, Natick, MA, 2019 b). Data are presented as median and 25th–75th percentile [Q1;Q3] unless reported otherwise. Normality was tested using the Shapiro–Wilk test. A Spearman correlation test was used to assess the correlation between Pcrit and different anthropometric and PSG parameters. Paired *t* tests were performed to identify significant differences between the two Pcrit calculation methods (peak inspiratory vs. ventilation). Statistical significance was defined as *P* value ≤ 0.05.

## RESULTS

Data from 13 patients were included in this feasibility study. The baseline characteristics of the patients are summarized in Table 1. The study population included middle-aged, overweight men with a baseline AHI of 24.9 events/h [20.1;43.9], indicating moderate to severe OSA.

**Table 1.** Baseline characteristics

Parameter, *n* = 13	Median	[Q1;Q3]
Anthropometrics and demographics		
Men, *n*	13	
Age, yr	51	49;56
BMI, kg/m^2^	28.10	25.10;31.7
Weight, kg	88	82;97
Neck circumference, cm	42	40;43
Polysomnographic		
AHI, events/h	24.9	20.1;43.9
Obstructive AHI, events/h	24.5	19.9;43.4
Supine AHI, events/h	42.9	32.23;60.20
Nonsupine AHI, events/h	19.65	13.18;28.57
REM-AHI, events/h	26.3	13.2;46.0
NREM-AHI, events/h	24	19.0;39.4
Hypopnea index	22.50	19.9;29.3
Apnea index	1.2	0;4.70
ODI, events/h	18.45	9.90;28.30
Evaluations		
Pcrit_peak_†	−0.84	−2.07;0.69
Pcrit_vent_	−1.32	−2.32;0.47
Propofol, µg/mL	2.45	2.30;2.85
Rus_peak_, cmH_2_O/L/s	19.3	15.3;25.4
Pnolim, cmH_2_O	9.4	8.2;10.2
Pclose, cmH_2_O	0.3	−0.5;1.6
One series time, min	14	13;15
Three series time, min	51	49;60
ESS, score 0–24	7	4;9
VAS snoring, 0–10	7	6;7

† Pcrit_peak_ could not be reliably calculated in one subject. AHI, apnea-hypopnea index; BMI, body mass index; ESS, Epworth sleepiness scale; NREM, non-rapid eye movement sleep; ODI, oxygen desaturation index; Pclose, closing pressure; Pcrit_peak_, critical closing pressure defined using the peak inspiratory method; Pcrit_vent_, critical closing pressure defined using the ventilation method; Pnolim, minimally effective pressure level at which airflow limitation was first abolished; REM, rapid eye movement sleep; Rus_peak_, peak upstream resistance; VAS, visual analogue scale.

**Figure 5.**
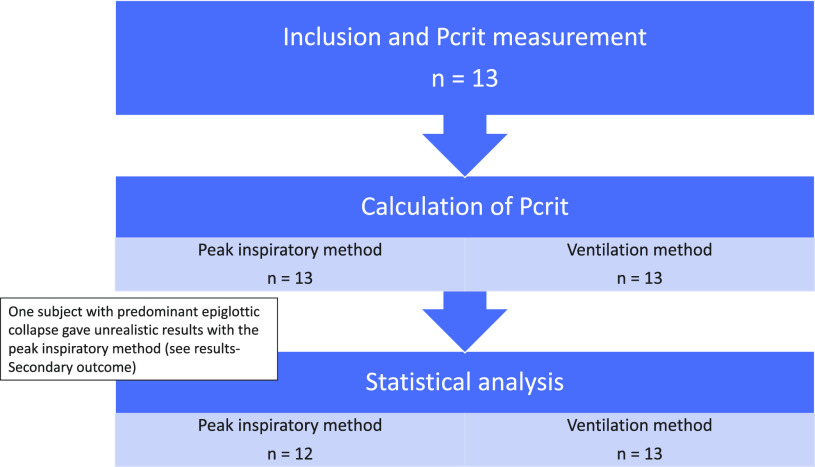
Flow chart of subjects for critical closing pressure (Pcrit) measurement and calculation.

A flowchart of the study is shown in Fig. 5. Pcrit was calculated in all 13 subjects using both the “ventilation method” and “peak inspiratory method.” One subject (*subject 7*) with predominant epiglottic collapse gave unrealistic results with the peak inspiratory method. Therefore, results of this patient were removed and only 12 subjects were used for further analysis using the peak inspiratory method. There was no baseline data on the oxygen desaturation index (ODI), neck circumference, and percentage of supine sleep in one subject.

### Primary Outcome

#### Pcrit-DISE feasibility.

The study showed that it is feasible to measure Pcrit during DISE. A routine clinical DISE takes ∼30–45 min. In this study, the median time interval between the start time of the pressure-drop procedure and finish time for three series in each subject was 51 [49–60] min and for one series was 14 [13–15] min. This period excludes the “onset of action” waiting period for the anesthesia medication that takes ∼10–15 min. The median propofol used in our study was 2.45 µg/mL [2.30;2.85].

**Figure 6.**
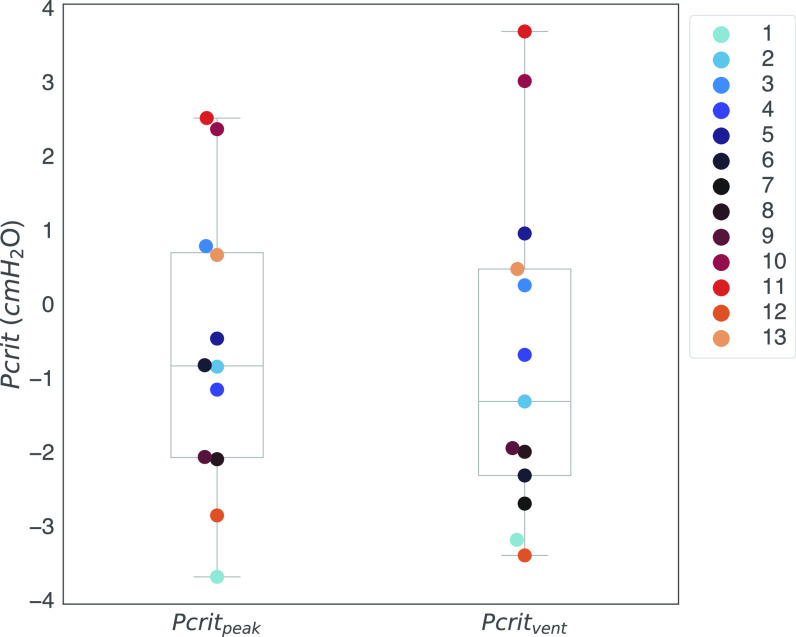
Critical closing pressure (Pcrit) as calculated with both methods [using peak inspiratory (Pcrit_Peak_) and inspiratory ventilation (Pcrit_Vent_)]. Note that *patient 7* gave unrealistic results using the peak inspiratory method and was therefore excluded for further analysis with this method. A color is assigned to each subject.

Multiple linear regression for repeated measures was performed to predict Pcrit based on pressure and peak inspiratory flow or inspiratory ventilation. A regression line could be fitted for both methods in the 12 subjects (Pcrit_peak_ *R*^2^ = 0.88, Pcrit_vent_ *R*^2^ = 0.82). Figure 6 shows the calculated Pcrit with the two different methods in all subjects and Table 2 shows the values for each subject. Median Pcrit_peak_ was −0.84 [−2.07; 0.69] cmH_2_O (*n* = 12) and median Pcrit_vent_ was −1.32 [−2.32;0.47] cmH_2_O (*n* = 13). Pairwise comparison showed no statistical difference in Pcrit between the different calculation methods (*P* = 0.67).

**Table 2.** Individual results for each patient including Pcrit, Rus, Pnolim, and Pclose

Subject	Pcrit_peak_ ± SE	Pcrit_vent_ ± SE	Rus_peak_ ± SE	Pnolim, cmH_2_O	Pclose, median
1	−3.69 ± 0.57	−3.19 ± 0.69	19.1 ± 1.8	7.2	−0.51
2	−0.85 ± 0.16	−1.32 ± 0.28	13.5 ± 0.4	6.6	−0.43
3	0.78 ± 0.80	0.25 ± 0.59	30.8 ± 3.3	8.4	2.4
4	−1.16 ± 0.31	−0.69 ± 0.43	25.9 ± 0.8	9.4	1.18
5	−0.47 ± 1.4	0.95 ± 0.42	30.7 ± 2.4	8.3	1.45
6	−0.83 + 0.38	−2.32 ± 0.24	9.4 ± 1.2	7.9	−0.62
7	−7.36 ± 1.64	−2.7 ± 0.44	30.6 ± 4.35	10	1.4
8	−2.1 ± 0.22	−2 ± 0.23	18.1 ± 0.5	10.2	−1
9	−2.07 ± 0.33	−1.95 ± 1.03	25.3 ± 1.6	9.5	-0.55
10	2.36 ± 0.4	3.01 ± 0.56	16.0 ± 1.0	10.3	2.65
11	2.51 ± 0.26	3.68 ± 0.35	19.5 ± 0.4	12.98	2.17
12	−2.86 ± 0.23	−3.4 ± 0.46	24.1 ± 0.8	9.52	−2.4
13	0.66 ± 0.25	0.47 ± 0.54	12.4 ± 0.7	13.3	1.04
Median	−0.84	−1.32	19.3	9.4	0.3
Q1;Q3	−2.07; 0.69	−2.32; 0.47	15.3; 25.4	8.2; 10.2	−0.5; 1.6

Pclose, closing pressure; Pcrit, critical closing pressure; Pcrit_peak_, Pcrit defined using the peak inspiratory method; Pcrit_vent_, Pcrit defined using the ventilation method; Pnolim, minimally effective pressure level at which airflow limitation was first abolished; Rus, upstream resistance defined as the reciprocal of the slope of the pressure-flow relationship.

**Figure 7.**
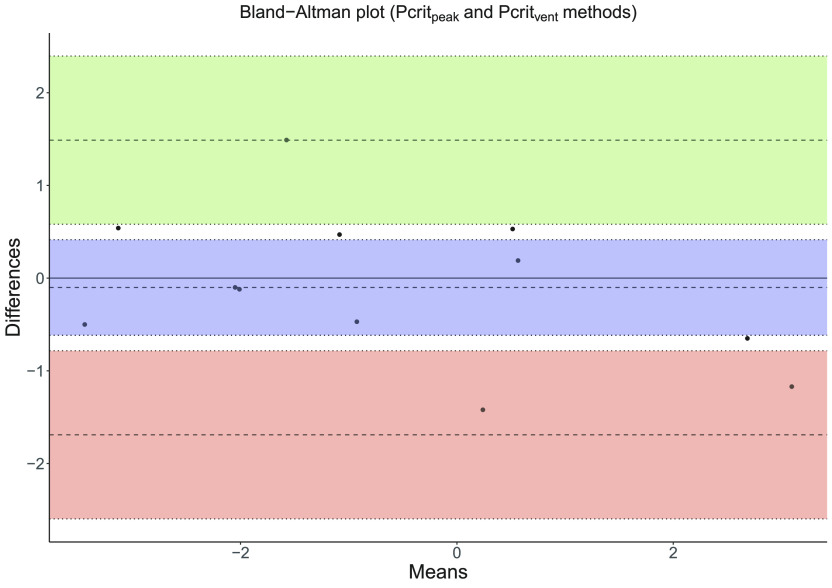
Bland–Altman plot for comparison of Pcrit_Peak_ and Pcrit_Vent_ methods showing within-pair means on *x*-axis and between-pair differences on *y*-axis (bias = −0.10, upper limit of agreement = 1.48, lower limit of agreement = −1.68, confidence interval = 95%). Pcrit_Peak_, critical closing pressure using peak inspiratory; Pcrit_Vent_, critical closing pressure using inspiratory ventilation.

Bland–Altman analysis showed that the Pcrit derived by the peak inspiratory method did not differ systematically from the Pcrit of the ventilation method and that there is no consistent bias of one approach versus the other (Fig. 7).

In one subject (*subject 7*, Table 2), there was a great discrepancy between both methods. Further inspection showed that the Pcrit calculation with “peak inspiratory method” gave strongly outlying results (−7.36 ± 1.64 cmH_2_O, *R*^2^ = 0.67). However, with the “ventilation method,” Pcrit was −2.72 ± 0.44 cmH_2_O (*R*^2^ = 0.73), within comparable ranges of other patients.

**Figure 8.**
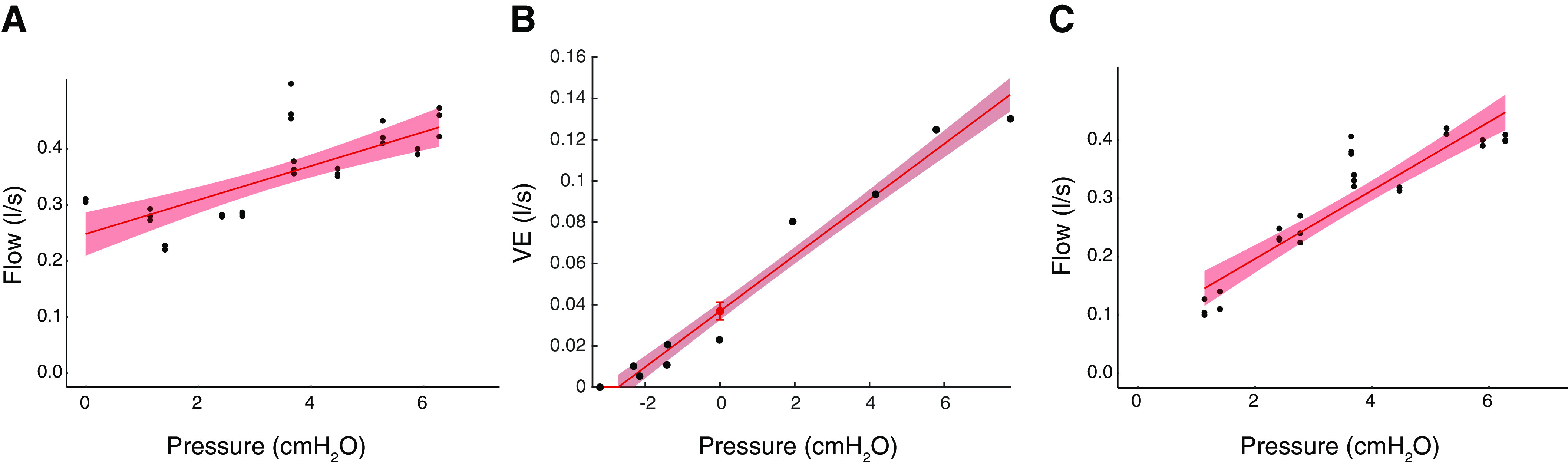
Representatives of pressure-flow linear regression of *subject 7* for all three calculation methods. *A*: peak inspiratory method (Pcrit = −7.36 cmH_2_O). *B*: V̇e, minute ventilation converted to l/s, ventilation method (Pcrit = −2.72 cmH_2_O). *C*: mid inspiratory method (Pcrit = −0.59 cmH_2_O)

It was found that this subject had predominant epiglottic collapse during DISE and that the airflow pattern pointed to the presence of high negative effort dependence (NED). NED is defined as the percentage reduction in inspiratory flow from peak to plateau and is associated with epiglottic collapse. Breaths with high NED have a high peak at the start of inspiration, followed by a low plateau inspiration, which can induce a bias toward higher than actual detected flow using the peak method (31–33). Figure 3*F* shows a section of this subject’s airflow recording. To overcome this short NED segment, the Pcrit for *subject 7* was also calculated using the following, flat effort-independent segment of inspiratory flow. This method resulted in a Pcrit of −0.59 ± 0.33 cmH_2_O (*R*^2^ = 0.88) (Fig. 8).

In another subject with epiglottic collapse, Pcrit with both methods was within a reasonable range for both methods (Pcrit_peak_ = −0.83 + 0.38 cmH_2_O and Pcrit_vent_ = −2.32 ± 0.24, *subject 6*, Table 2). However, the *R*^2^ of this subject’s regression model for Pcrit_peak_ was 0.55 and for Pcrit_vent_ was 0.79, indicating a better fit of the ventilation method.

### Secondary Outcomes

#### Pressure-flow relationship outcomes.

The upstream resistance (Rus), defined as the reciprocal of the slope of the pressure-flow relationship, had a median of 19.3 cmH_2_O/L/s [15.3;25.4]. The median of the minimally effective CPAP level at which airflow limitation was first abolished (Pnolim) was 9.4 [8.2;10.2] cmH_2_O. The median level of the nasal pressure at which inspiratory airflow was first ceased as observed during the measurements (Pclose-observed) was 0.3 [−0.5;1.6] cmH_2_O.

There was a significant correlation between Pclose-observed and both Pcrit_peak_ (*r* = 0.81, *P* = 0.002) and Pcrit_vent_ (*r* = 0.88, *P* = 0.0001). However, there was no significant correlation between the Pnolim and any of the calculated Pcrits via the two methods or Pclose-observed. Furthermore, there was no correlation between Rus and any of the Pnolim, Pclose-observed, Pcrit_vent_, and Pcrit_peak_.

#### Anthropometric/polysomnographic parameters and Pcrit.

There was no significant correlation between any of the anthropometric or polysomnographic variables and Pcrit, Rus, and Pclose-observed (Table 3). Age was the only parameter that showed a significant negative correlation with Pnolim (*r* = −0.63, *P* = 0.02).

**Table 3.** Spearman correlation coefficients of the relationship between anthropometric and polysomnographic variables, and Pcrit and Rus, Pnolim, and Pclose

	Pcrit_peak_		Pcrit_vent_		Rus_peak_		Pnolim		Pclose	
	Rho	*P*	Rho	*P*	Rho	*P*	Rho	*P*	Rho	*P*
Age, yr	−0.19	0.55	−0.27	0.36	−0.04	0.87	−0.63	0.02	−0.21	0.49
BMI, kg/m^2^	0.13	0.68	0.14	0.62	0.16	0.61	0.24	0.44	−0.18	0.59
Neck circumference, cm	0.06	0.84	−0.01	0.95	−0.36	0.26	−0.24	0.46	−0.04	0.88
AHI, events/h	−0.12	0.69	−0.09	0.74	−0.26	0.40	−0.02	0.95	−0.30	0.33
Apnea index, events/h	−0.16	0.61	−0.03	0.89	0.02	0.94	−0.04	0.87	−0.16	0.61
Hypopnea index, events/h	−0.15	0.63	−0.11	0.72	−0.30	0.34	−0.06	0.83	−0.32	0.29
ODI, events/h	0.07	0.83	−0.06	0.83	−0.13	0.69	−0.07	0.83	−0.11	0.73
Supine AHI, events/h	0.09	0.79	0.35	0.25	0.39	0.23	−0.34	0.29	0.25	0.45

AHI, apnea-hypopnea index; BMI, body mass index; ODI, oxygen desaturation index; Pclose, closing pressure; Pcrit, critical closing pressure; Pcrit_peak_, Pcrit defined using the peak inspiratory method; Pcrit_vent_, Pcrit defined using the ventilation method; Pnolim, minimally effective pressure level at which airflow limitation was first abolished; Rus, peak upstream resistance.

#### DISE pattern and Pcrit.

**Figure 9.**
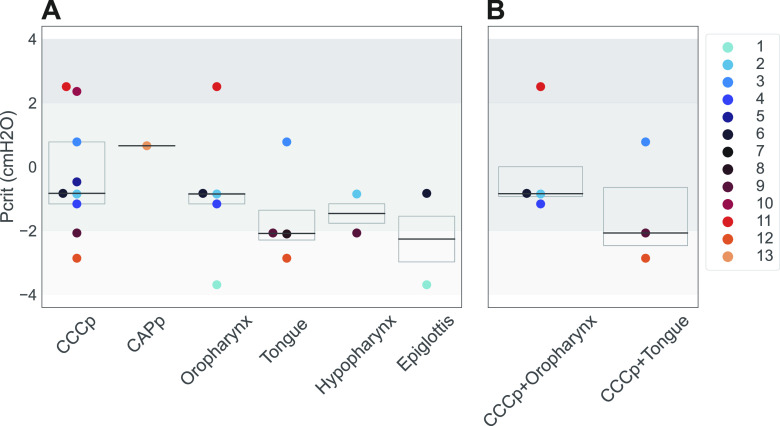
Drug-induced sleep endoscopy (DISE) findings and critical closing pressure (Pcrit) values. *A*: palatal complete concentric collapse (CCCp), palatal complete anteroposterior collapse (CAPp), collapse at the level of oropharynx, tongue base, hypopharynx, and epiglottis. *B*: CCCp in combination with collapse at the level of oropharynx or tongue base. A color is assigned to each subject.

Next, the site(s) and pattern(s) of collapse during DISE were evaluated (Fig. 9). Complete concentric collapse at the soft palate (CCCp), which is associated with several non-CPAP treatment outcomes (8, 16), was present in nine subjects (69.2%). These subjects showed a median Pcrit of −0.83 cmH_2_O, ranging from a minimum of −2.86 cmH_2_O to a maximum of 2.51 cmH_2_O. Subjects with combined CCCp and tongue base collapse had a median Pcrit of −2.07 cmH_2_O [−2.46;−0.64], *P* = 0.44, whereas subjects with combined CCCp and oropharyngeal collapse exhibited a median Pcrit of −0.84 cmH_2_O [−0.92;0], *P* = 0.8. Table 4 summarizes the frequency of each collapse pattern and its associated Pcrit data.

**Table 4.** Summary of the frequency of each collapse type and their associated Pcrit_peak_ data

	Median Pcrit_peak_	[Q1;Q3]	Min Pcrit	Max Pcrit	% Collapse Frequency
CCCp	−0.83	−1.16;0.78	−2.86	2.51	69.23
CAPp	0.66	0.66;0.66	0.66	0.66	7.69
Oropharynx	−0.85	−1.16;−0.83	−3.69	2.51	38.46
Tongue	−2.08	−2.29;−1.56	−2.86	0.78	30.76
Hypopharynx	−1.46	−1.76;−1.15	−2.07	−0.85	15.38
Epiglottis	−2.26	−2.97;−1.54	−3.69	−0.83	23.07
CCCp + oropharynx	−0.84	−0.92;0	−1.16	2.51	30.76
CCCp + tongue	−2.07	−2.46;−0.64	−2.86	0.78	23.07

One Pcrit value was calculated in each subject and the collapse pattern was evaluated via drug-induced sleep endoscopy (DISE). [Q1;Q3], quantile 1;quantile 3. CAPp, palatal complete anteroposterior collapse; CCCp, palatal complete concentric collapse.

## DISCUSSION

Pcrit is a measure of collapsibility of the pharyngeal airway during sleep (18). DISE provides information about the level, degree, and configuration (pattern/direction) of the upper airway collapse in subjects with OSA (12, 13). This clinical trial was the first to investigate the feasibility and protocol of Pcrit measurement during routine clinical DISE. The proposed protocol was successful in measuring Pcrit during routine clinical DISE and the results were in accordance with the available results in literature (19, 21, 25, 34–38). Each measurement took approximately 1 h for three repeated series of pressure drop. Each series took ∼15 min (45 min for 3 series). In addition, 15 min for “onset of action” waiting period for the anesthesia medication was taken into account. There was no significant difference between the Pcrit calculated using peak inspiratory or inspiratory ventilation method, which confirms the reliability of these measurements. The ventilation method was, however, more successful in assessment of Pcrit in subjects with epiglottis collapse, which is commonly associated with high NED (31, 32). Subjects with CCCp during DISE had a wide range of Pcrit from −2.86 to 2.51 cmH_2_O, suggesting no direct association between this specific DISE phenotype and Pcrit.

The results of the current clinical trial indicate that Pcrit measurement was feasible during DISE without any complications, allowing concomitant measurement of collapsibility and site, degree, and direction of the upper airway collapse. Several studies have previously measured Pcrit in healthy subjects and in subjects with OSA under midazolam-induced sleep, but without endoscopy (28, 39–43). Previously, to develop a mathematical model of the upper airway, CPAP was used to change the upper airway orifice size under varying pressures while measuring the neutral cross-sectional area with endoscopy under propofol sedation (30). These studies emphasized the facilitating role of sedation in overcoming the recurring arousals that inherently occur with pressure drops during natural sleep. Sedation shortens the period of time needed for measurements in contrast to natural sleep. An added advantage of Pcrit measurement during drug-induced sleep is the possibility to perform these measurements during daytime working hours and eliminate the need for an overnight study. It provides an opportunity for Pcrit measurements to not only be bound to research environments but also could possibly be performed in routine practice in a fast-paced environment with high patient turnover.

DISE assists therapy selection and is routinely performed in many of the patients with OSA who seek CPAP alternatives. These non-CPAP therapies have proven to be more effective in upfront selected subjects (8, 15–17). In phenotypic models of subjects with OSA, it has been shown that subjects with a Pcrit ≥ +2 cmH_2_O respond better to major anatomic and mechanical interventions such as CPAP, whereas those with a Pcrit ≤ +2 cmH_2_O might benefit from other therapies such as MAD, surgical interventions, medications, or a combination of these targeted therapies (11). Other studies have shown that collapsibility is associated with non-CPAP treatment outcomes (7, 8, 44). Therefore, incorporation of Pcrit measurements into DISE assessments might give valuable additional information for the management and follow-up of the subjects (7, 8, 44).

In this and previous studies, multiple pressure drop series were performed. To evaluate the potential for further time reduction in Pcrit assessment during DISE, the minimum required series of pressure drops to achieve a reliable Pcrit was explored. Comparison of Pcrit, as measured by using the data of the three series of pressure-drop repetitions with the data of only one series, showed no statistically significant difference (*P* = 0.07). Therefore, it might be possible to further reduce the duration of the measurement of Pcrit during DISE by only performing one series of pressure drop and further enhance the possibility to perform this measurement in routine clinical practice.

A particular strength of the present study was using two different calculation methods for Pcrit. The calculation of Pcrit with the two different methods showed no statistically significant difference and proved that both calculation methods are reliable (Pcrit_peak_ *R*^2^ = 0.88, Pcrit_vent_ *R*^2^ = 0.82). In *subject 7* with predominant epiglottic collapse during DISE, the peak inspiratory method was unable to show reliable Pcrit results, whereas the ventilation method gave a reliable value (Pcrit_peak_ *R*^2^ = 0.67, Pcrit_vent_ *R*^2^ = 0.73). This might be explained by the breath shape of subjects with epiglottic collapse (32, 33). Overall, these breaths are characterized by a steep, high-peak inspiratory flow and a sudden drop in the inspiratory airflow as a result of the sudden clasp-knife-like closure of the epiglottis, defined as a high NED (31–33). Therefore, in these cases, using the peak inspiratory airflow for Pcrit calculations is not recommended as it can lead to interpreting the breaths as having no flow limitation and thus false low Pcrit values. This was also confirmed by the noticeable difference of *R*^2^ for the two methods in another subject (*subject 6*) with epiglottic collapse (Pcrit_peak_ *R*^2^ = 0.55, Pcrit_vent_ *R*^2^ = 0.79) while this difference was smaller for patients without epiglottic collapse (median Pcrit_peak_ *R*^2^ = 0.91, Pcrit_vent_ *R*^2^ = 0.83, all subjects excluding *subjects 7* and *6*). Using the midinspiratory plateau flow is another way to overcome the pinching effect that is present in breaths with high NED. To assess this possibility, the Pcrit with midinspiratory airflow was also calculated for *subject 7* (midinspiratory Pcrit = −0.59 ± 0.33 cmH_2_O, *R*^2^ = 0.88). Considering the standard errors and *R*^2^ of these three methods (Pcrit_peak_ *R*^2^ = 0.67, Pcrit_vent_ *R*^2^ = 0.73, Pcrit mid-inspiratory *R*^2^ = 0.88), the flat, effort-dependent method, and the ventilation method are preferred in *subject 7*.

The main advantage of the Pcrit_vent_ method is that it is able to capture both simple patterns (“flat-top”) and complex patterns (“discontinuities,” “scooping”) of inspiratory flow limitation; complex patterns are more common than appreciated and are pervasive in patients with certain sites of collapse (epiglottic). Furthermore, the use of ventilation to describe airflow provides a practical and semiautomated approach for characterizing the upper airway mechanics and is simply achieved by integrating the inspiratory flow signal. We, therefore, consider that the ventilation method allows for a more unified or robust method to calculate the critical closing pressure without the need for additional tailoring of the method for each patient. Furthermore, the ventilation-v-pressure curve value itself reflects the ventilation that is achievable under different CPAP pressures; we argue that this is of great importance as it reflects whether sufficient ventilation can be achieved for gas exchange and metabolic demand.

A limitation of the Pcrit_vent_ method is the effect of the inspiratory duration (Ti). Ti rises with pharyngeal obstruction (prolonged filling time with lower elastic recoil acting to terminate inspiration) and thus could protect against the detection of obstruction. However, we note that this effect, if of a meaningful magnitude, would also be revealed as a curvilinear ventilation-v-pressure relationship, yet the method provided a greater *R*^2^ than that for peak flow-v-pressure in patients with epiglottic collapse (see results). Therefore, the ventilation method allows for a more linear relationship with pressure in certain patients who preserve their peak flow despite the development of progressive obstruction and lowered ventilation (i.e., those with epiglottic collapse); the increased linearity was thereby considered to increase the validity of the resultant critical closing pressure values. We also note that even in theory, the increased Ti cannot have any impact at the *x*-intercept, i.e., at the critical collapsing pressure when the airway is fully collapsed; notably, this is the value reported in the current analysis.

“Pclose-observed” was 0.3 [−0.5;1.6] cmH_2_O. There was a significant correlation between Pclose-observed and both Pcrit_peak_ (*r* = 0.81, *P* = 0.002) and Pcrit_vent_ (*r* = 0.88, *P* = 0.0001). Pclose-observed showed to be a surrogate for assessment of collapsibility in subjects with OSA. This finding confirms that the level of collapsibility can be assessed during DISE and as such highlights that the pathophysiological traits of site of upper airway collapse and collapsibility can be measured concomitantly (20). Important to note is the different definitions for Pclose as reported in literature. In earlier papers, Pclose is used instead of the term Pcrit for critical closing pressure (45). Furthermore, Isono (46) defined the Pclose based on a mathematical model of the pressure-area relationships of the passive pharynx in anesthetized, completely paralyzed subjects. These definitions are not to be confused with the concept of “Pclose-observed” in our study, which is based on median level of the nasal pressure at which inspiratory airflow was first ceased as observed during the measurements (20, 47).

In the current study, a nasal mask was used for measurements while a surgical paper tape was fixed over the lips and mouth to prevent mouth breathing and air leak. Previous research showed that mouth breathing and an oronasal mask would cause a bias in the measured airflow and pressure due to the posterior displacement of tongue, which leads to a decrease in the upper airway patency (48, 49). In general, comparison of a nasal mask with other masks has shown a systematically higher Pcrit and therapeutic CPAP with oronasal or full-face masks (48, 50, 51). A nasal mask is recommended as it reduces the possible surface area for air leaks and allows air to only enter through the nasal cavity. Therefore, the use of a nasal mask and the rigorous attention for mouth leaks throughout the research protocol is a clear strength of the current study.

This study has several limitations. The current study population consisted of middle-aged, overweight men with moderate to severe OSA. Previous studies have shown a propensity for higher collapsibility levels with higher body mass index (BMI) and neck circumference (19, 52). One study showed that with the increase in age, there is a slight increase in collapsibility (0.6 cmH_2_O/decade) (53). However, in this study, Pcrit showed no significant correlation with any of the anthropometric or PSG parameters. This might indicate that Pcrit is an independent parameter contributing to OSA and points to the added value of determining Pcrit in patients with OSA. However, considering the rather small sample size, future studies with a larger patient population are needed to confirm these findings.

Another interesting observation was present in subjects with CCCp on DISE. Subjects with CCCp and subjects with higher collapsibility levels have common endotypic OSA characteristics (8, 16, 54, 55). Therefore, it is assumed that subjects with CCCp might have high levels of collapsibility. Contrary to expectations, subjects with CCCp in this study had a wide range of measured Pcrit (from −2.86 to 2.51 cmH_2_O). A possible explanation for these results may be that correlating CCCp with Pcrit is an oversimplification of the complicated underlying pathophysiology of CCCp and OSA. There might not be a direct correlation between collapsibility, measured by Pcrit, and the site, pattern, and degree of upper airway collapse, indicating these are two independent traits, each contributing to OSA. However, as discussed, our study subjects were mainly middle-aged overweight men, which are the typical characteristics for both CCCp and high collapsibility (56). Moreover, CCCp was present in nine out of 13 subjects, which is a high proportion compared with other studies (16, 28, 39–43). Our subjects had a rather high supine AHI (42.3/h [2.23;60.20]) on their baseline PSG and the measurements were exclusively performed in supine position; hence this elevated supine AHI might partially explain the high prevalence of CCCp among our subjects. In addition, in the subjects with CCCp, any lower-level obstruction could be a secondary collapse due to the negative pressures generated following the primary obstruction at the level of the velopharynx. In future research, the use of a multilevel pressure catheter might shine more light on this hypothesis. Therefore, validation of these results in a larger OSA cohort is needed.

In literature, a variety of medications for sedation during DISE are described. However, target-controlled infusion of propofol is the method that has proven resemblance to upper airway collapse pattern and collapsibility of natural sleep (57, 58). In addition, studies have shown that collapsibility levels during midazolam sedation are similar to that of natural sleep (34). According to Heiser et al. (59), the level of sedation is achieved when the brain concentration of propofol is ∼3 μg/mL. The propofol pump can be programmed to 2 or 2.5 μg/mL, with an increase of 0.5 μg/mL every 2 min until the level of sedation is achieved. Furthermore, an addition of 1–2 mg of midazolam at the beginning of the sedation will shorten the time to the observation window (13, 59, 60). Therefore, in the current study, a combination of target-controlled infusion of propofol and midazolam was opted for induction of sleep. However, these sedative medications might have influenced the severity of collapsibility, as it was previously shown that an increase in depth of anesthesia via application of different concentrations of propofol increases collapsibility (61). In another study, a nonlinear relationship between the loss of consciousness and elevation of collapsibility has been shown (62). This study concluded that the increase in collapsibility with the loss of consciousness might be due to the abrupt reduction of genioglossus muscle activity while transitioning from awake to sedation (62). However, in a previous study by Genta et al. (28), there was no significant difference between Pcrit measured during natural sleep and drug-induced sleep using midazolam. To avoid this effect, in the present study, measurements were performed after a stable level of anesthesia was achieved and the propofol concentration was not modified during the measurement. Therefore, we cannot evaluate the effect of a change in propofol concentration on collapsibility. Future studies are needed to investigate this effect.

The passive Pcrit measurement protocol mimics the abrupt reduction of genioglossus muscle activity while transitioning from awake to sedation (62). Based on this finding, passive Pcrit measurements might be preferred over active Pcrit measurement in a setting involving anesthesia. In addition, as a result of the limitations of time during measurements under sedation and the time-consuming nature of active Pcrit measurements, the present study reports the passive but not the active Pcrit. It would be interesting to measure active Pcrit during DISE in another investigation.

Another potential limitation of this study is the inherent limitation of endoscopy, where not all collapse sites can be visualized simultaneously. This was tackled by looking at a different level of the upper airway during each of the three series of pressure drop and reporting the combination of collapse levels in each of the subjects, as well as each individual collapse level. However, it is possible that secondary collapse was present.

Furthermore, another limitation of the present study is the limited sample size and the absence of gender diversity. Further studies with the current set-up and a larger, more diverse sample size are required to validate the results of the current investigation. Finally, the current study showed the need for an expert analysis when it comes to evaluation of airflow and pressure signals. Currently, this is a manual process, future research is needed to automate this process.

### Conclusions

To our knowledge, this clinical study was the first to investigate the feasibility and protocol of Pcrit measurement during DISE, allowing simultaneous determination of the collapsibility and the site of upper airway collapse. The proposed protocol of this clinical study was successful in measurement of Pcrit during DISE and the results were in accordance with previous results in literature. Calculation of measured Pcrit was reliable, showing no statistically significant difference between the two different calculation methods using peak inspiratory flow or ventilation. However, the ventilation method was more successful in assessment of Pcrit in subjects with epiglottic collapse, which is typically associated with higher NED. Contrary to expectations, subjects with CCCp had a wide range of measured Pcrit (from −2.86 to 2.51 cmH_2_O). This finding may suggest that the surmised correlation between a higher Pcrit and the presence of CCCp could be an oversimplification of the complicated underlying pathophysiology of CCCp and OSA. Incorporation of Pcrit measurements into the DISE assessments is feasible and might give valuable additional information for the next step in the management of OSA.

## GRANTS

M.D. holds a senior postdoctoral fellowship at the Research Foundation Flanders (FWO) (12H4520N). S.A.S. was supported by the National Institute of Health-National Heart, Lung, and Blood Institute (R01HL146697) and the American Academy of Sleep Medicine (AASM) Foundation (228-SR-20). O.M.V. holds a Senior Clinical Fellowship Grant (Fundamenteel Klinisch Mandaat) from the Research Foundation Flanders – Vlaanderen (FWO) [1833517N]. S.O.d.B. holds a postdoctoral fellowship at the Research Foundation Flanders (FWO) (1299822N). 

## DISCLOSURES

J.V. reports grants from SomnoMed, AirLiquide, Vivisol, Mediq Tefa, Medidis, OSG, Bioprojet, Desitin, Philips, and ResMed outside the submitted work. S.A.S. reports consulting fees from Apnimed, Nox Medical, Merck, and Eli Lilly unrelated to the current work, plus unrelated grant support from Apnimed, Prosomnus, and Dynaflex; his outside activities are actively monitored by his institution. O.M.V. reports grants from Philips and Somnomed at Antwerp University Hospital and others from Inspire Medical Systems, Nightbalance, GSK, and Liva Nova at Antwerp University Hospital outside the submitted work. No conflicts of interest, financial or otherwise, are declared by the other authors. 

## AUTHOR CONTRIBUTIONS

E.K., O.M.V., and S.O.d.B. conceived and designed research; E.K., E.V.d.P., M.W., and S.O.d.B. performed experiments; E.K. and S.O.d.B. analyzed data; E.K., M.D., O.M.V., and S.O.d.B. interpreted results of experiments; E.K. and S.O.d.B. prepared figures; E.K. and S.O.d.B. drafted manuscript; E.K., E.V.d.P., M.D., M.W., J.V., S.A.S., O.M.V., and S.O.d.B. edited and revised manuscript; E.K., E.V.d.P., M.D., M.W., J.V., S.A.S., O.M.V., and S.O.d.B. approved final version of manuscript.
